# Supplementary material for: Uncovering the Contribution of Moderate-Penetrance Susceptibility Genes to Breast Cancer by Whole-Exome Sequencing and Targeted Enrichment Sequencing of Candidate Genes in Women of European Ancestry
Source: Cancers (Basel). 2022 Jul 11;14(14):3363. doi: 10.3390/cancers14143363 (PMC9317824; doi:10.3390/cancers14143363)
Supplement: Supplementary file 1 [file cancers-14-03363-s001.zip › Figure S1-final_16052022.pptx]

## Slide 1
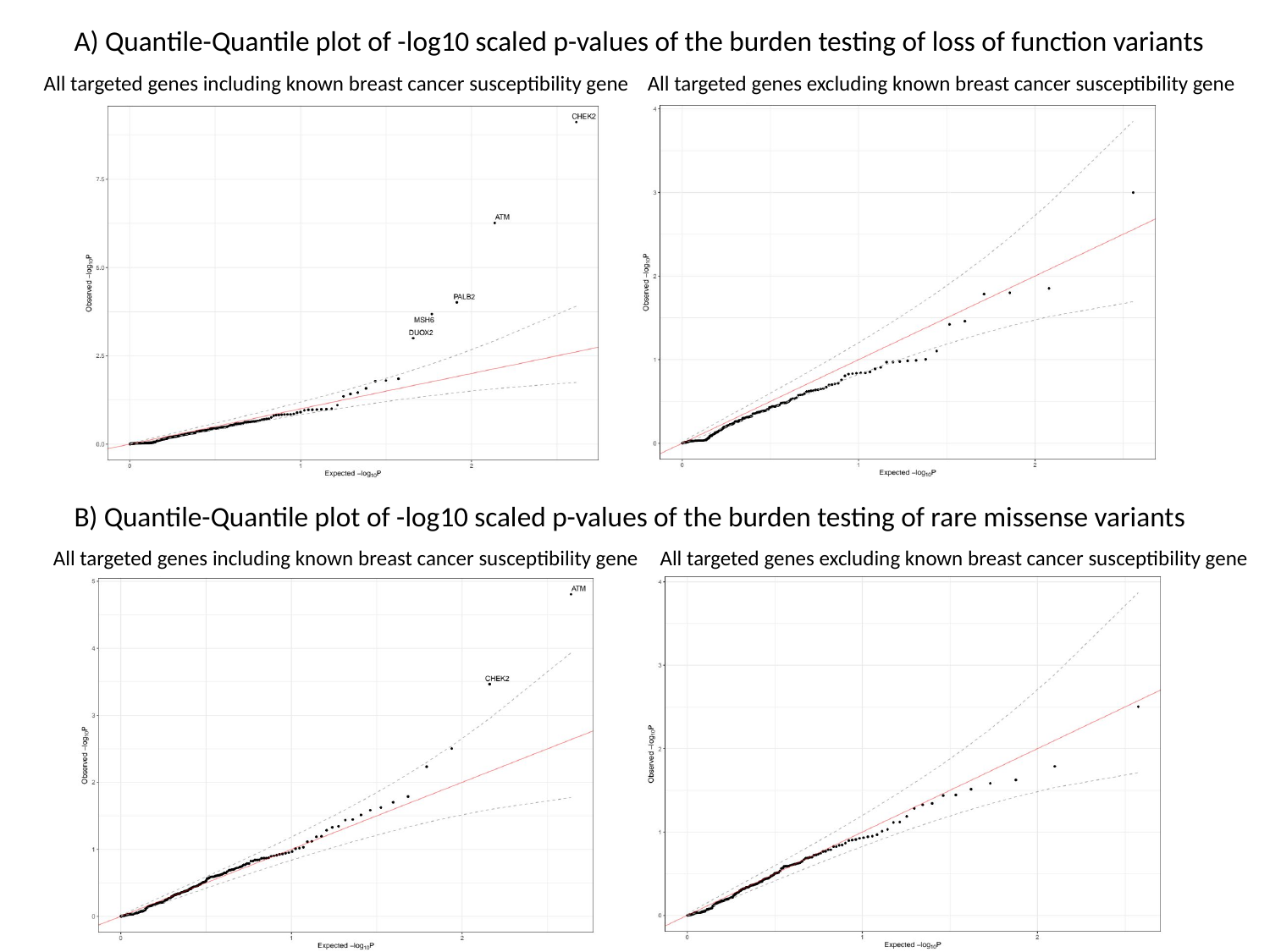

A) Quantile-Quantile plot of -log10 scaled p-values of the burden testing of loss of function variants
All targeted genes including known breast cancer susceptibility gene
All targeted genes excluding known breast cancer susceptibility gene
8
6
4
2
0
0.0
0.5
1.0
1.5
2.0
B) Quantile-Quantile plot of -log10 scaled p-values of the burden testing of rare missense variants
All targeted genes including known breast cancer susceptibility gene
All targeted genes excluding known breast cancer susceptibility gene
