# Supplementary material for: Uncovering the Contribution of Moderate-Penetrance Susceptibility Genes to Breast Cancer by Whole-Exome Sequencing and Targeted Enrichment Sequencing of Candidate Genes in Women of European Ancestry
Source: Cancers (Basel). 2022 Jul 11;14(14):3363. doi: 10.3390/cancers14143363 (PMC9317824; doi:10.3390/cancers14143363)
Supplement: Supplementary file 1 [file cancers-14-03363-s001.zip › Supplementary materials_revised_07072022_clean.pdf]

SUPPLEMENTARY MATERIALS

Supplementary Methods

Figure S1. Quantile-quantile plot of the observed vs. expected chi-squared statistics for A) loss-of-function and B) missense variants in the analysis of 230 target genes using logistic regression adjusted for the origin cohort. Each circle represents the chi-squared statistic for a gene. The red diagonal line represents the predicted association statistics under the global null hypothesis of no association.

Table S1: Description of studies included in the discovery and validation analyses.

Table S2: Selection of genes for the validation stage according to gene prioritization strategies.

Table S3: List of A) loss-of-function variants and B) rare missense variants identified in overall breast cancer at the validation stage.

Table S4: List of A) loss-of-function variants and B) missense variants identified in ER-negative breast cancer cases at the validation stage.

Table S5: List of A) loss-of-function variants and B) missense variants identified in ER-positive breast cancer cases at the validation stage.

Table S6: Associations, by study, of loss-of-function and rare missense variants in 32 genes included in commercial breast cancer susceptibility gene panels and breast cancer risk. OR denotes odds ratio and CI, confidence interval.

Table S7: Associations of rare missense variants in 32 genes included in commercial breast cancer gene panels and breast cancer risk overall (6,211 cases, 6,019 controls), ER-negative (808 cases, 6,019 controls) and ER-positive breast cancer risk (2,764 cases, 6,019 controls). OR denotes odds ratio and CI, confidence interval.

Table S8: Associations of A) loss-of-function variants, B) rare missense variants, and C) combined loss of function and rare missense variants in 178 genes identified at the discovery stage (6,211 cases, 6,019 controls) with overall breast cancer risk and ER-negative and ER-positive breast cancer risk. OR denotes odds ratio and CI, confidence interval.

Table S9: Associations with breast cancer risk of loss-of-function and rare missense variants in 178 genes identified at the discovery stage, by study. OR denotes odds ratio and CI, confidence interval.

Table S10: Associations of loss-of-function and rare missense variants in 178 genes identified at the discovery stage with A) ER-negative breast cancer risk (808 cases, 6,019 controls) and B) ER-positive breast cancer risk (2,764 cases, 6,019 controls). OR denotes odds ratio and CI, confidence interval.

Table S11: Associations of individual A) loss of function and B) rare missense variants, observed in at least 3 carriers among breast cancer cases and controls that included at least 2 breast cancer cases, and overall breast cancer

Funding

References

Supplementary Methods

Discovery Stage – WES

Breast cancer studies

The DFBBCS study includes *BRCA1/2* mutation negative familial bilateral breast cancer patients selected from five clinical genetics centers, namely Erasmus University Medical Center/Daniel den Hoed, The Netherlands Cancer Institute, Leiden University Medical Center, University Medical Center Utrecht, and VU University Medical Center [1-3]. All subjects from DFBBCS have bilateral breast cancer. A total of 511 breast cancer cases were drawn from the DFBBCS study. 2) The GC-HBOC study group comprises 16 university centers that collect data on families suspected of having hereditary breast and ovarian cancer based on a defined set of clinical ascertainment criteria. Subjects include families with clustering or early onset of breast or ovarian cancer and tested for the absence of deleterious germline mutations in *BRCA1* and *BRCA2*. All subjects tested negative for the pathogenic founder variant c.1100del in *CHEK2*. Subjects did not have a personal history of ovarian cancer. Study details have been described previously [4]. For the discovery stage, a total of 1,021 breast cancer cases were specifically drawn from the GC-HBOC centers of Munich or Cologne.

Control datasets

The data generated from whole-exome sequencing of the breast cancer cases have been analysed with those of available control exome datasets.

Data from controls matched to the DFBBCS study were drawn from two studies: The Rotterdam Study and The Genome of the Netherlands Project (GoNL) study. The Rotterdam Study is a single center population-based follow-up study conducted by the Erasmus University Medical Center. The main objective is to investigate the prevalence and incidence of risk factors for chronic diseases. The rationale and design of the study has been described in detail elsewhere [5]. As of 2008, 14,926 subjects aged 45 years or over comprised the cohort. Baseline measurements were obtained between 1990 and 1993. All participants were subsequently examined in follow-up examination rounds every 2-3 years. Whole-exome sequence data was obtained for 2,625 samples. Data was generated using NimbleGen SeqCap EZ exome V2 with a mean coverage of 55× (44 Mb capture). GoNL is led by a consortium comprising five university centers [6]. The project has sequenced the whole genomes of 250 Dutch parent-offspring families with an intermediate coverage in sequencing depth of ~13× [7]. Whole-exome data extracted from whole-genome sequencing was obtained for 500 unrelated individuals. Data extracted included 300 bp padded capture regions from the following: NimbleGen SeqCap EZ exome V2, NimbleGen SeqCap EZ exome V3, Agilent SureSelect Human All Exome V5 (194 Mb capture).

Data from controls matched to the GC-HBOC study samples have been drawn from the KORA-Study and the German Study on Ageing, Cognition, and Dementia (AgeCoDe). AgeCoDe is a prospective longitudinal study on the early detection of mild cognitive impairment and dementia in general practice. The study was conducted at six German cities (Bonn, Duesseldorf, Hamburg, Leipzig, Mannheim, and Munich). Between January 1, 2003 and November 30, 2004, a total of 3,327 subjects free of dementia at baseline were recruited and assessed with structured clinical interviews and cognitive tests. In 2016, follow-up 9 was completed. More study details have been previously described elsewhere [8]. Whole-exome data was obtained for 396 samples. Data was generated using NimbleGen SeqCap EZ exome V2 (44 Mb capture). The KORA-Study is a population-based biobank from Southern Germany comprising about 18,000 individuals aged between 25-74 years at recruitment. Follow-up investigations are performed at regular intervals. Information is available on socio-demography, environmental factors, nutrition, smoking,

general medical history such as disease and use of medication. The study has been described in detail elsewhere [9]. Whole-exome data was obtained for 209 samples. Data was generated using Agilent SureSelect Human All Exon V3 technology (50Mb target size) with a mean coverage of 90×.

Library preparation and high-throughput sequencing

Library preparation and high-throughput sequencing were performed at two centers: CHU de Quebec – Université Laval Research Center, Quebec City, Canada and McGill University and Genome Quebec Innovation Center, Montreal, Canada. For samples prepared using the Agilent SureSelect XT Human All Exon V5 (Agilent Technologies, Santa Clara, USA), 1.5 g of genomic DNA (gDNA) was fragmented on a Covaris instrument (Covaris, Woburn, MA, USA). Fragments of 150-200bp were purified and checked for quality control using either a Tapestation 2200 (Agilent Technologies, Santa Clara, USA) or a LabChip GX (Perkin Elmer, Woodridge, Canada) instrument. Library preparations were completed using standard sample preparation protocol: end repair, adenylation, ligation of paired-end adaptors. After adaptor ligation and purification, libraries were amplified for 13 cycles in the pre-capture amplification step. Following hybridization to the target-specific capture library and purification using streptavidin-coated beads, libraries were amplified for 14 cycles with indexing primers. A final QC was completed to check library size (250–350 bp).

For samples prepared using the NimbleGen SeqCap EZ Exome Library v3.0 at a 4-plex, between 100-500ng of gDNA were fragmented on a Covaris instrument (Covaris, Woburn, MA, USA). Fragmented DNA were used to perform end repair, A-tailing and adapter ligation with KAPA library preparation kits (Kapa Biosystems Inc. Wilmington, MA, USA), following the manufacturer instructions. Quality controls using a LabChip GX (Perkin Elmer, Woodridge, Canada) instrument were performed as requested by the protocol. Libraries were amplified for 7 cycles in the pre-capture amplification step. Subsequently, pools of 4 amplified indexed libraries were hybridized the target-specific capture baits, recovered using streptavidin-coated beads and amplified for 17 cycles. A final QC was completed to check library size (150–400 bp).

Bioinformatics analysis

All computations were run on Calcul Québec supercomputer Colosse. Samples were demultiplexed with bcl2fastq 1.8.4 (Illumina), trimmed with Trimmomatic v0.32 [10] then aligned to the reference genome (hg19) using BWA-MEM v0.7.10 [11] to create aligned BAM-files, followed by duplicate reads tagging and Base Quality Score Recalibration using Picard MarkDuplicates v1.123 (Broad Institute) and GATK v3.2 [12]. Variant calling per sample was performed using a pipeline based on GATK Haplotype Caller in GVCF mode, then the whole sample set was subjected to joint-calling with GATK GenotypeGVCFs. Calls were refined with GATK VariantRecalibrator, in order to compute their scores. AnnoVar [13] was used to annotate the callset with NCBI refGene informations, variants frequencies (from 1000 Genomes [14], Exome Server Project 6500 (NHLBI GO Exome Sequencing Project) and *in-silico* variants deleteriousness scores (from dbnsfp30a [15]).

Variant Filtering and Gene Prioritization Strategies

University of Cambridge (Strategy 1)

Quality filtering was performed on variants following ExAC standards [16]. Subsequently, filtered variants were annotated with VEP [17] and Loftee (Loss-Of-Function Transcript Effect Estimator) [18] which enabled to focus on loss of function (LoF) variants. Results were then prioritized by computing per gene odds ratios, on genes having LoF variants in cases and in controls.

Munich Technical University (Strategy 2)

Variants were filtered using genotype quality scores and sequencing depth. Then, annotation was applied with AnnoVar [13] and a kinship matrix was computed, both within EPACTS (Efficient and Parallelizable Association Container Toolbox, University of Michigan). To prioritize the resulting genes, filtered variants were separated into four groups: LoF variants, very rare missense variants (<1%), rare missense variants (1-5%), and a combination of rarest LoF and missense variants (<1%). On all groups, burden tests were performed, and variants were annotated with ExAC [16] counts, presence in COSMIC [19], and presence in breast cancer genes pathways.

CHU de Québec - Université Laval Research Centre (Strategy 3)

Variant filtering was performed, on minimal supporting depth, and on a different cut-off on variant genotype quality scores for single nucleotide variants and small insertions or deletions. Then, variants were annotated using AnnoVar [13], which enabled the filtering on variants deleteriousness (namely SIFT [20], PolyPhen-2 [21], and CADD Phred scores [22], as well as on variant frequency (ESP6500 (NHLBI GO Exome Sequencing Project), 1000 Genomes [14], and ExAC [19] to keep the most deleterious and rare variants. Gene prioritization was based on CADD Phred scores. Each gene received a sum of all CADD Phred scores of the variants within, then the sum was normalized using the gene coding sequence size.

Huntsman Cancer Institute, University of Utah (Strategy 4)

A complete software suite called VICTOR (Variant Interpretation for Clinical Testing Or Research) [23] was used to annotate and quality-control variants. Filtration on the genotype level was based on genotype quality and read depth. Filtration on the variant level was based on variant quality scores, with different thresholds for single nucleotide variants and small insertions or deletions. Variants were also filtered on calling missing rate, calling missing rate discrepancy between cases and controls, Hardy-Weinberg equilibrium. The pipeline also included gene prioritization suite PERCH (Polymorphism Evaluation, Ranking and Classification for Heritable traits), which ranks genes by quantitatively integrating deleteriousness prediction, allele frequency information, rare variant association analysis, biological relevance assessment, and the quality of variant calls.

University of Toronto (Strategy 5)

Variants were filtered on quality parameters. The "ActiveDriver" method was used for gene prioritization. This method identifies "active" sites in proteins that are specifically and significantly mutated in cancer genomes. Four types of post-translational modification sites (phosphorylation, ubiquitination, acetylation, methylation) were evaluated [24]. ActiveDriver was performed separately on cases and controls. Genes with significantly more mutations flagged by ActiveDriver in cases than in controls were ranked.

Validation Stage – targeted enrichment sequencing

Breast cancer case-control studies

The GC-HBOC study has been described in the previous section. Of note that different samples from those used in the discovery set were used in the validation set. The Dutch validation sample set was comprised of two studies, the Amsterdam Breast Cancer Study - Familial (ABCS-F) [1] and the Rotterdam Breast Cancer Study (RBCS) [2]. ABCS-F is a clinical genetic center-based case study. Non-*BRCA1/2* breast cancer cases were recruited from the family cancer clinic of the NKI-AVL in the period 1995-2009. Cases were diagnosed with breast cancer during 1965-2012. RBCS is a hospital-based case-control study from the Rotterdam area. Familial breast cancer patients were selected from the Clinical Genetics Center at Erasmus MC Cancer Institute; recruitment periods ranging from 1994 - 2005 (RBCS1) and 1995 - 2009 (RBCS2). Controls were drawn from a population-based cohort of women from all ages recruited through the Sanquin blood bank. Studies of Epidemiology and Risk factors in Cancer Heredity (SEARCH) is a

population-based study with breast cancer cases ascertained through the Eastern Cancer Registration and Information Centre (ECRIC) [25]. The study enrolled subjects diagnosed before age 55 years with invasive breast cancer from 1991 onwards and who were still alive at the start of the study in 1996, together with all patients diagnosed before age 70 years after 1996. Controls were drawn from three sources: (1) general practices participating in SEARCH who were frequency matched by age to the cases; (2) the European Prospective Investigation of Cancer (EPIC)-Norfolk study, a population-based cohort study of diet and health in Norfolk, East Anglia [26] and (3) women undergoing breast screening as part of the National Health Service Breast Screening Programme in screening centres in Cambridgeshire, who participated in the Sisters in Breast Screening study [27]. For the Ontario Familial Breast Cancer Registry (OFBCR) invasive cases were identified from the Ontario Cancer Registry during1996-1998. Cases diagnosed during 1996-1998 were identified from the Ontario Cancer Registry which registers >97% of all cases residing in the province at the time of diagnosis. Invasive cases aged 20–54 years who met the OFBCR definition for high genetic risk (family history of specific cancers particularly breast and ovarian, early onset disease, Ashkenazi ethnicity or a diagnosis of multiple breast cancer). A 25% random sample of individuals in this age category who did not meet the OFBCR definition, 35% of those aged 55–69 at high risk and 8.75% aged 55–69 at low risk were also asked to participate. Individuals diagnosed in 2001 and 2002 were also included if they met the high-risk criteria. Unrelated, unaffected population controls were recruited during 2003-2005 by calling randomly selected residential telephone numbers throughout the same geographical region. Eligible controls were women with no history of breast cancer and were frequency-matched by 5-year age group to the expected age distribution of cases. [28]. CARTaGENE (CaG) (www.cartagene.qc.ca) is a population-based biobank designed to investigate social, environmental and medical determinants of chronic diseases. The CaG study is comprised of individuals aged 40–69 years from the province of Québec (Canada). Details of the study have previously been published [29]. Briefly, participants were randomly selected between July 2009 to October 2010 from the population of four metropolitan areas in Quebec (Montreal, Québec City, Sherbrooke, Saguenay) using the governmental health insurance database for identification. Data were collected from over 20,000 individuals, which represents 1% of the 40–69 years old population of the province of Québec.

Library preparation and high-throughput targeted sequencing

Libraries were prepared using the Agilent SureSelect QXT target Enrichment kit (Agilent Technologies, Santa Clara, USA). 50ng of gDNA were enzymatically fragmented and adaptors were added to ends of the fragments in a single reaction. Libraries were amplified for 8 cycles in the pre-capture amplification step using adaptors primers. Following hybridization to the target-specific capture library and purification using streptavidin-coated beads, libraries were amplified for 12 cycles with indexing primers. A final QC using a Tapestation 220 (Agilent Technologies, Santa Clara, USA) was completed to check library size (325–450 bp).

Target intervals were created from the union of the custom bait's "Covered" intervals (Agilent 3034901.zip document) and the coding exons of the 230 target genes padded by 2bp on each side. Coding exons were retrieved from the UCSC table export (https://genome.ucsc.edu/cgibin/hgTables) using assembly hg19, track NCBI RefSeq.

Bioinformatic analysis

BAM files were generated using a pipeline based GATK Best Practices that included: trimming using Trimmomatic [10], alignment using BWA-MEM [11] against b37 human genome with decoys, indel realignment using GATK 3.2.2, [12] fixing mate coordinate using samtools and bvatools, marking duplicates using Picard v 1.123 MarkDuplicate (Broad Institute), base quality recalibration using GATK 3.2.2 [12], and metrics generation and collection. Genomic VCF were generated using HaplotypeCaller

from GATK 4.1.0.0 and following GATK's best practices. Joint calling was performed using GATK 4.1.0.0 and Cromwell v. 34 using the best practice workflow joint-discovery-gatk4-local from gatk4-germline-snps-indels. The VCF was further processed using VICTOR (Version 1.2beta Build 2019-03-19) [23] against VICTOR's GRCh37 databases (v. 2018-06-12 updated 2018-12-14). VICTOR performs various steps of quality controls and filtering, both at the locus and sample level. Sample level quality control included high locus missing rate amongst samples, low mean GQ, and low mean DP. A first locus-based filtering was performed on the basis of non-genic status, low VQSLOD score, high missing rate, failed FILTER field, missing rate significantly different between cases and controls, and quality hard filter thresholds. A second locus-based filtering was performed using functional annotation where variants were filtered on the following basis: synonymous variations, non-coding variants (intron, 3'-UTR, 5'-UTR), or a reported clinical significance of 'benign'. A final filtering was performed based on low prediction of deleteriousness either based on the BayesDel score or on the reported allele frequency (MaxAF<0.01). In addition, filtering was performed to remove technical duplicates and population outliers of non-European ancestry. Samples with loss of function (LoF) variants in *BRCA1* and *BRCA2* were also removed. Loss of function variants in the last exon were removed except for single exon genes. When multiple variants were observed within 6 bp of each other in the same individual, these variants were considered to be in linkage disequilibrium and only one variant was considered in the variant counts.

A list of LoF and missense variants identified at the validation stage are listed in Table S3 (overall breast cancer), Table S4 (ER-negative breast cancer) and Table S5 (ER-positive breast cancer).

Funding

The **PERSPECTIVE** project was supported by the Government of Canada through Genome Canada and the Canadian Institutes of Health Research (GPH-129344), the Ministère de l'Économie, de la Science et de l'Innovation du Québec through Genome Quebec, and the Quebec Breast Cancer Foundation. The PRE[3]VENTION project was supported by a grant from the Ministère de l'Économie, de la Science et de l'Innovation du Québec through the PSR-SIIRI-949 program. **GC-HBOC:** The German Consortium for Hereditary Breast and Ovarian Cancer (GC-HBOC) is funded by the German Cancer Aid (#110837, #70111850) and the Federal Ministry of Education and Research (BMBF), Germany (grant no 01GY1901). Next-generation sequencing of female case and control individuals was supported by the Ministry for Innovation, Science and Research of the State of North Rhine- Westphalia (#323-8.0302.16.02-132142). LIFE Leipzig Research Center for Civilization Diseases, Universität Leipzig, is funded by means of the European Union, by the European Regional Development Fund (ERDF), and by means of the Free State of Saxony within the framework of the excellence initiative. **The Rotterdam Study:** The Rotterdam Study is funded by Erasmus Medical Center and Erasmus University, Rotterdam, Netherlands Organization for the Health Research and Development (ZonMw), the Research Institute for Diseases in the Elderly (RIDE), the Ministry of Education, Culture and Science, the Ministry for Health, Welfare and Sports, the European Commission (DG XII), and the Municipality of Rotterdam. The generation and management of genomics data for the Rotterdam Study is supported by the Netherlands Organisation of Scientific Research NWO Investments (nr. 175.010.2005.011, 911-03-012) and the Netherlands Genomics Initiative (NGI)/NWO project nr. 050-060-810 (Netherlands Consortium for Healthy Ageing; NCHA). **GoNL:** Funding for the project was provided by the Netherlands Organization for Scientific Research under award number 184021007, dated July 9, 2009 and made available as a Rainbow Project of the Biobanking and Biomolecular Research Infrastructure Netherlands (BBMRI-NL). **KORA-Study Group:** The KORA research platform (KORA, Cooperative Research in the Region of Augsburg) was initiated and financed by the Helmholtz Zentrum München – German Research Center for Environmental Health, which is funded by the German Federal Ministry of Education and Research and by the State of Bavaria. Furthermore, KORA research was supported within the Munich Center of Health Sciences (MC Health), Ludwig-Maximilians-

Universität, as part of LMUinnovativ. **SEARCH:** SEARCH is funded by a programme grant from Cancer Research UK (C490/A10124) and supported by the UK National Institute for Health Research Biomedical Research Centre at the University of Cambridge. **OFBCR** was supported by grant UM1 CA164920 from the National Cancer Institute (USA). **CARTaGENE:** The CARTaGENE study was supported by Genome Canada.

References

1. Schmidt, M.K.; Hogervorst, F.; van Hien, R.; Cornelissen, S.; Broeks, A.; Adank M.A.; Meijers, H.; Waisfisz, Q.; Hollestelle, A.; Shutte, M.; et al. Age- and Tumor Subtype-Specific Breast Cancer Risk Estimates for CHEK2*1100delC Carriers. *J Clin Oncol* **2016**, *34*, 2750-2760. doi: 10.1200/JCO.2016.66.5844.

2. Kriege, M.; Hollestelle, A.; Jager, A.; Huijts, E.M.; Berns, E.M.; Sieuwerts, A.M.; Meijer-van Gelder, M.E.; Collée, J.M.; Devilee, P.; Hooning, M.J.; et al. Survival and contralateral breast cancer in CHEK2 1100delC breast cancer patients: impact of adjuvant chemotherapy. *Br J Cancer* **2014**, *111*, 1004-1013. doi: 10.1038/bjc.2014.306.

3. Schmidt, M.K.; Tollenaar, R.A.; de Kemp, S.R.; Broeks, A.; Cornelisse, C.J.; Smit V.T.; Peterse, J.L.; van Leeuwen F.E.; Van't Veer, L.J. Breast cancer survival and tumor characteristics in premenopausal women carrying the CHEK2*1100delC germline mutation. *J Clin Oncol* **2007**, *25*, 64-69. doi: 10.1200/JCO.2006.06.3024.

4. Kast, K.; Rhiem, K.; Wappenschmidt, B.; Hahnen, E.; Hauke, J.; Bluemcke, B.; Zarghooni, V.; Herold, N.; Ditsch, N.; Kiechle, M.; et al. Prevalence of BRCA1/2 germline mutations in 21 401 families with breast and ovarian cancer. *J Med Genet* **2016**; *53*, 465–471. doi: 10.1136/jmedgenet-2015-103672.

5. Ikram, M.A.; Brusselle, G.; Ghanbari, M.; Goedegebure, A.; Ikram, M.K.; Kavousi, M.; Kieboom, B.C.T.; Klaver, C.C.W.; de Knegt, R.J.; Luik, A.I.; et al. Objectives, design and main findings until 2020 from the Rotterdam Study. *Eur J Epidemiol* **2020** *35*, 483-517. doi: 10.1007/s10654-020-00640-5.

6. Boomsma, D. I.; Wijmenga, C.; Slagboom, E. P.; Swertz, M. A.; Karssen, L. C.; Abdellaoui, A.; Ye, K.; Guryev, V.; Vermaat, M.; van Dijk, F.; et al. The Genome of the Netherlands: design, and project goals. *European Journal of Human Genetics* **2013**, *22*, 221-227. doi:10.1038/ejhg.2013.118.

7. Genome of the Netherlands Consortium. Whole-genome sequence variation, population structure and demographic history of the Dutch population. *Nature Genetics* **2014**, *46,* 818-825. doi: 10.1038/ng.3021.

8. Jessen, F.; Wiese, B.; Bickel, H.; Eiffländer-Gorfer, S.; Fuchs, A.; Kaduszkiewicz, H.; Köhler, M.; Luck, T.; Mösch, E.; Pentzek, M.; et al. Prediction of dementia in primary care patients. *PLoS One* **2011**, *6*, e16852. doi: 10.1371/journal.pone.0016852.

9. Wichmann, H-E.; Gieger, C.; Illig, T.; MONICA/KORA Study Group. KORA-gen-resource for population genetics, controls and a broad spectrum of disease phenotypes. *Gesundheitswesen* **2005**, *67*, S26-30. doi: 10.1055/s-2005-858226.

10. Bolger, A.M.; Lohse, M.; Usadel, B. Trimmomatic: A Flexible Trimmer for Illumina Sequence Data. *Bioinformatics* **2014,** *30*, 2114–2120. doi: 10.1093/bioinformatics/btu170.

11. Li, H. Aligning sequence reads, clone sequences and assembly contigs with BWA-MEM. *ArXiv: Genomics* **2013**, *1303.3997*.

12. McKenna, A.; Hanna, M.; Banks, E.; Sivachenko, A.; Cibulskis, A.; Kernytsky, A.; Garimella, K.; Altshuler, D.; Gabriel, S.; Daly, M.; et al. The Genome Analysis Toolkit: A MapReduce Framework for Analyzing Next-Generation DNA Sequencing Data. *Genome Res* **2010**, *20*, 1297–1303. doi: 10.1101/gr.107524.110.

13. Wang, K.; Li, M.; Hakonarson, H. ANNOVAR: Functional Annotation of Genetic Variants from High-Throughput Sequencing Data. *Nucleic Acids Res* **2010**, *38*, e164. doi: 10.1093/nar/gkq603.

14. 1000 Genomes Project Consortium; Auton, A.; Brooks, L.D.; Durbin, R.M.; Garrison, E.P.; Kang, H.M.; Korbel, J.O.; Marchini, J.L.; McCarthy, S.; McVean, G.A.; et al. A Global Reference for Human Genetic Variation. *Nature* **2015**, *526*. 68–74. doi: 10.1038/nature15393.

15. Liu, X.; Wu, C.; Li, C.; Boerwinkle, E. dbNSFP V3.0: A One-Stop Database of Functional Predictions and Annotations for Human Nonsynonymous and Splice-Site SNVs. *Hum Mutat* **2016**, *37*, 235–241. doi: 10.1002/humu.22932.

16. Lek, M.; Karczewski, K.J.; Minikel, E.V.; Samocha, K.E.; Banks, E.; Fennell, T.; O'Donnell-Luria, A.H.; Ware, J.S.; Hill, A.J.; Cummings, B.B.; et al. Analysis of Protein-Coding Genetic Variation in 60,706 Humans. *Nature* **2016**, *536*, 285–291. doi: 10.1038/nature19057.

17. McLaren, W.; Gil, L.; Hunt, S.E.; Riat, H.S.; Ritchie, G.R.S.; Thormann, A.; Flicek, P.; Cunningham, F. The Ensembl Variant Effect Predictor. *Genome Biol* **2016**, *17*, 122. doi: 10.1186/s13059-016-0974-4.

18. Karczewski, K.J.; Francioli, L.C.; Tiao, G.; Cummings B.B.; Alföldi, J.; Wang, Q.; Collins, R.L.; Laricchia, K.M.; Ganna, A.; Birnbaum, D.P.; et al. The mutational constraint spectrum quantified from variation in 141,456 humans. *Nature* **2020**, *581*, 434–443. doi: 10.1038/s41586-020-2308-7.

19. Forbes, S.A.; Beare, D.; Gunasekaran, P.; Leung, K.; Bindal, N.; Boutselakis, H.; Ding, M.; Bamford, S.; Cole, C.; Ward, S.; et al. COSMIC: Exploring the World's Knowledge of Somatic Mutations in Human Cancer. *Nucleic Acids Res* **2015**, *43*, D805-811. doi: 10.1093/nar/gku1075.

20. Kumar, P.; Henikoff, S.; Ng, P.C. Predicting the Effects of Coding Non-Synonymous Variants on Protein Function Using the Sift Algorithm. *Nat Protoc* **2009**, *4*, 1073–1081. doi: 10.1038/nprot.2009.86.

21. Adzhubei, I.; Jordan, D.M.; Sunyaev, S.R. Predicting Functional Effect of Human Missense Mutations Using Polyphen-2. *Curr Protoc Hum Genet* **2013**, *7*, unit 7.20. doi: 10.1002/0471142905.hg0720s76.

22. Kircher, M.; Witten, D.M.; Jain, P.; O'Roak, B.J.; Cooper, G.M.; Shendure, J. A General Framework for Estimating the Relative Pathogenicity of Human Genetic Variants. *Nat Genet* **2014**, *46*, 310–315. doi: 10.1038/ng.2892.

23. Feng, B-J. PERCH: A Unified Framework for Disease Gene Prioritization. *Hum Mutat* **2017**, *38*, 243–251. doi: 10.1002/humu.23158.

24. Reimand, J.; Bader, G.D. Systematic Analysis of Somatic Mutations in Phosphorylation Signaling Predicts Novel Cancer Drivers. *Mol Syst Biol* **2013,** *9*, 637. doi: 10.1038/msb.2012.68.

25. Dunning, A.M.; Healey, C.S.; Pharoah, P.D.; Teare M.D.; Ponder, B.A.; Easton, D.F. A systematic review of genetic polymorphisms and breast Cancer risk. *Cancer Epidemiol Biomarkers Prev* **1999**; *8*: 843–854.

26. Day, N.; Oakes, S.; Luben, K.; Khaw, K.T.; Bingham, S.; Welch, A.; Wareham, N. EPIC-Norfolk: study design and characteristics of the cohort. European prospective investigation of Cancer. *Br J Cancer* **1999**, *80*, 95–103.

27. Kataoka, M.; Antoniou, A.; Warren, R.; Leyland, J.; Brown, J.; Audley, T.; Easton, D. Genetic models for the familial aggregation of mammographic breast density. *Cancer Epidemiol Biomarkers Prev* **2009**, *18*, 1277–1284. doi: 10.1158/1055-9965.EPI-08-0568.

28. John, E.M.; Hopper, J.L.; Beck, J.C.; Knight, J.A.; Neuhausen, S.L.; Senie, R.T.; Ziogas, A.; Andrulis I.L.; Anton-Culver, H.; Boyd, N.; et al. The Breast Cancer Family Registry: an infrastructure for cooperative multinational, interdisciplinary and translational studies of the genetic epidemiology of breast cancer. *Breast Cancer Res* **2004**, *6*, R375–R389. doi: 10.1186/bcr801.

29. Awadalla, P.; Boileau, C.; Payette, Y.; Idaghdour, Y.; Goulet, J-P.; Knopper B.; Hamet, P.; Laberge, C.; CARTaGENE Project. Cohort profile of the CARTaGENE study: Quebec's population-based biobank for public health and personalized genomics. *Int J Epidemiol* **2013**, *42*, 1285–1299. doi: 10.1093/ije/dys160.
