# Supplementary material for: Uncovering the Contribution of Moderate-Penetrance Susceptibility Genes to Breast Cancer by Whole-Exome Sequencing and Targeted Enrichment Sequencing of Candidate Genes in Women of European Ancestry
Source: Cancers (Basel). 2022 Jul 11;14(14):3363. doi: 10.3390/cancers14143363 (PMC9317824; doi:10.3390/cancers14143363)
Supplement: Supplementary file 1 [file cancers-14-03363-s001.zip › Table S1-revised_final_23062022.pdf]

Table S1. Description of studies and datasets included in the discovery and validation stages.

Study	Country	Cases (n)	Controls (n)	Study design	Case definition	Control definition	Selected familial cases	Capture technology	References
**Discovery step: whole-exome sequencing**									
**Breast cancer samples sets**									
German Consortium for Hereditary Breast & Ovarian Cancer (GC-HBOC)	Germany	1,021	-	Clinic-based case study	Women diagnosed with breast cancer in the GC-HBOC centres of Cologne or Munich from families with clustering or early onset breast or ovarian cancer. Recruitment period 1996-2015.	N/A	Yes	Agilent SureSelect Human All Exon V5	[4]
Dutch Familial Bilateral Breast Cancer Study (DFBBCS)	Netherlands	511	-	Clinic-based case study	Familial bilateral breast cancer cases diagnosed between 1962 and 2012 and selected from five clinical genetics centers, namely Erasmus University Medical Center, The Netherlands Cancer Institute, Leiden University Medical Center, University Medical Center Utrecht, and VU University Medical Center.	N/A	Yes	NimbleGen SeqCap EZ Exome Library v3.0	[1-3]
**Control datasets**									
KORA-Study Group	Germany	-	209	Population-based biobank	N/A	Population-based study conducted in Augsburg and the surrounding counties. Age range of the participants was 25 to 74 years at time of recruitment	-	Agilent SureSelect Human All Exon V3	[9]
AgeCoDe	Germany	-	396	Prospective longitudinal study	N/A	Subjects older than 75 years old from Germany were recruited between January 2003 and November 2004.	-	NimbleGen SeqCap EZ exome V2	[8]
GoNL	Netherlands	-	500	Biobank; whole-genome-	N/A	The parents from parent–offspring trios,	-	NimbleGen SeqCap EZ exome V2,	[6]

				sequencing project in a representative sample consisting of 250 trio-families from all provinces in the Netherlands		ranging in age from 19 to 87 from birth cohorts 1910–1994.		NimbleGen SeqCap EZ exome V3, Agilent SureSelect Human All Exome V5	
Rotterdam study	Netherlands	-	2,628	Population-based study	N/A	Prospective cohort study with recruitment of individuals from the city of Rotterdam over 45 years of age.	-	NimbleGen SeqCap EZ Exome Library v2.0	[5]
**Validation phase: targeted sequencing**									
German Consortium for Hereditary Breast & Ovarian Cancer (GC-HBOC)	Germany	3,199	2,767	Clinic-based case study	Women diagnosed with breast cancer in 16 GC-HBOC centres from families with clustering or early onset breast or ovarian cancer. Recruitment period 1996-present. The selected cases did not have a personal history of ovarian cancer. Recruitment period from March 2017 to September 2017. All cases previously tested negative for pathogenic BRCA1/2 germline variants and the pathogenic founder variant c.1100del in CHEK2.	Healthy, unrelated, ethnically and age-matched female control individuals (LIFE study, Leipzig, Germany). Samples and data were collected from December 2016 to August 2017. All individuals were at least 40 years old (mean age 63, range 40–92 years).	Yes	Agilent SureSelect QXT target Enrichment kit	[4]
Dutch Validation Sample Set: Amsterdam Breast Cancer Study - Familial (ABCS-F) and Rotterdam Breast Cancer Study (RBCS)	Netherlands	979	962	Clinical genetic center-based cases; Hospital-based case-control study, Rotterdam area	Familial breast cancer cases selected from two studies: ABCS-F cases: All non-BRCA1/2 breast cancer cases from the family cancer clinic of the NKI-AVL tested in the period 1995-2009; all ages and diagnosed with breast cancer in 1965-2012; RBCS cases: Cases were selected from the Clinical Genetics Center at Erasmus University Medical Center; recruited 1994 - 2009.	Population-based cohort of women from all ages recruited through the Sanquin blood bank.	Yes	Agilent SureSelect QXT target Enrichment kit	[1,2]

Ontario Familial Breast Cancer Registry (OFBCR)	Canada	600	591	Population-based familial case-control study	Cases diagnosed between 1996-1998 were identified from the Ontario Cancer Registry. Invasive cases aged 20–54 years who met the OFBCR definition for high genetic risk (family history of specific cancers particularly breast and ovarian, early onset disease, Ashkenazi ethnicity or a diagnosis of multiple breast cancer).	Unrelated, unaffected population controls were women with no history of breast cancer and were frequency-matched by 5-year age group to the expected age distribution of cases.	Yes	Agilent SureSelect QXT target Enrichment kit	[28]
Study of Epidemiology and Risk factors in Cancer Heredity (SEARCH)	UK	1,289	1,348	Population-based case-control study	Cases ascertained through the East Anglian Cancer Registry; 1) prevalent cases diagnosed 1991-1996 under 55 years of age at diagnosis, recruited 1996-2002; 2) incident cases diagnosed since 1996 under 70 years of age at diagnosis, recruited 1996- present.	Three sources of controls: (1) selected from the EPIC-Norfolk cohort study of 25,000 individuals age 45-74 recruited between 1992 and 1994, based in the same geographic region as cases; (2) selected from general practices from March 2003 to present, frequency matched to cases by age and geographic region; (3) women undergoing breast screening as part of the National Health Service Breast Screening Programme, who participated in the Sisters in Breast Screening Study	No	Agilent SureSelect QXT target Enrichment kit	[25-27]
Cartagene (CaG)	Canada	451	469	Population-based biobank	Self-reported cases were aged 40–69 years selected between July 2009 to October 2010 from the population of four metropolitan areas in Quebec using the governmental health insurance database for identification.	Healthy, unrelated, ethnically and age-matched female control individuals	No		[29]
